# Bilateral Common Iliac Artery Aneurysm, a Case Report

**DOI:** 10.21980/J83S73

**Published:** 2020-01-15

**Authors:** Laura Kolster, Danielle Biggs, Amy Patwa, Michael Gerardi

**Affiliations:** * Morristown Medical Center, Department of Emergency Medicine, Morristown, NJ

## Abstract

Patient is a 72-year-old male who presented with abdominal pain and hypertension. Through bedside point-of-care ultrasound (POCUS), patient was found to have markedly enlarged bilateral iliac artery aneurysms with signs of impending rupture, which was confirmed on computed tomography (CT) angiogram of the abdomen/pelvis. Patient was immediately taken to the operating room for emergent repair of aneurysm, and was discharged from the hospital the next day. Point-of-care ultrasound is critical in diagnosis and guiding treatment in patients in the emergency department and should be considered in those with undifferentiated abdominal pain.

**Topics:**

Abdominal pain, iliac artery aneurysm, point-of-care ultrasound.

[Fig f1-jetem-5-1-v8] [Fig f2-jetem-5-1-v8] [Fig f3-jetem-5-1-v8]

**Figure f1-jetem-5-1-v8:**
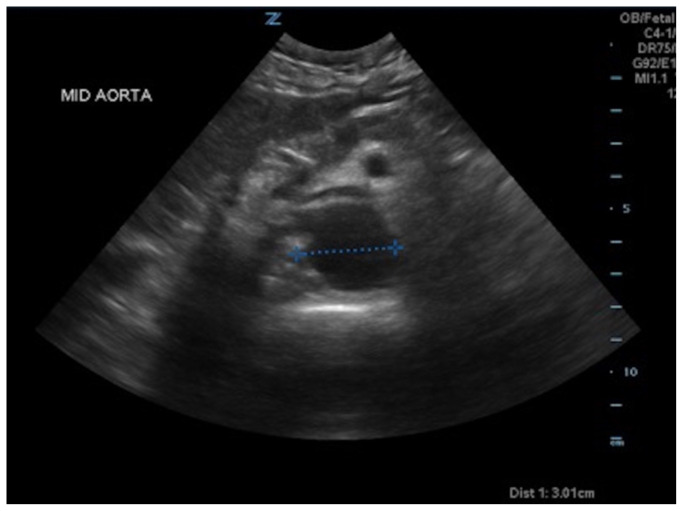


**Figure f2-jetem-5-1-v8:**
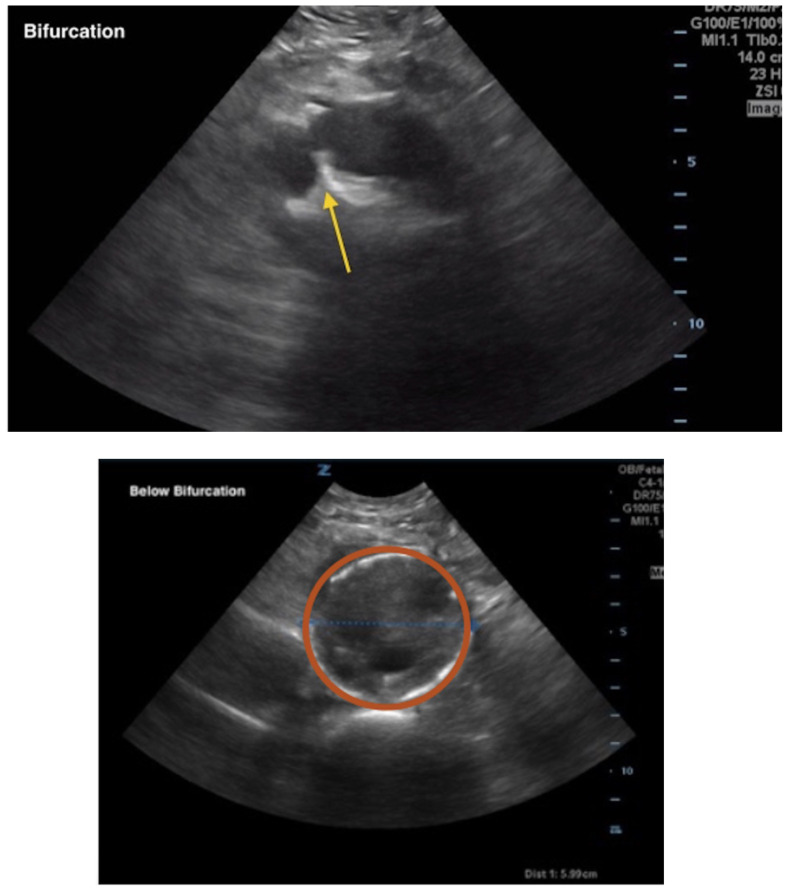


**Figure f3-jetem-5-1-v8:**
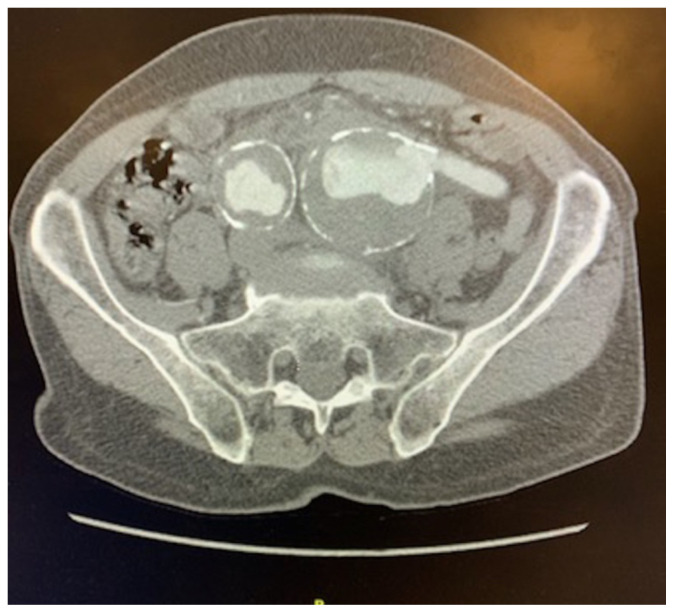
Video Link: https://youtu.be/SO8CJWeaERk

## Introduction

The role of point of care ultrasound for aortic aneurysm has been well outlined; however, the utility of iliac artery point of care ultrasound is not well defined. There is utility in obtaining ultrasound of bilateral iliac arteries because it can require immediate management in the operating room.

## Presenting concerns and clinical findings

Patient is a 72-year-old male who presents with a one-day history of constant, dull, periumbilical abdominal pain. The pain is associated with a decrease in appetite. Patient denies any fever, chills, vomiting, diarrhea, back pain, or dysuria. Patient had elective inguinal hernia surgery performed one year ago, with no other prior abdominal surgeries.

## Significant findings

A bedside ultrasound of the aorta was performed. The proximal, middle, and distal aorta appeared normal in caliber, as demonstrated by the images; however there seemed to be some enlargement at the bifurcation. The bifurcation into the iliac arteries, as highlighted by the yellow arrow, demonstrates a slightly enlarged iliac artery on the left. The aorta was followed below the bifurcation as it divided into the iliac arteries, as shown in the video clip. The ultrasound demonstrated a left iliac artery aneurysm measuring 5.99 cm, as highlighted by the orange circle. There were aneurysms to the bilateral common and internal iliac arteries.

## Patient course

On arrival the patient was found to be hypertensive with a blood pressure of 198/132. On exam he exhibited mild tenderness to palpation in the periumbilical region, and his abdomen was mildly distended. His distal pedal pulses were 2+ bilaterally. The rest of the exam was unremarkable.

The surgical team was immediately consulted. Since the patient was hemodynamically stable, a CT angiogram (CTA) of the abdomen and pelvis was performed. The CTA showed marked induration of the common iliac artery aneurysms suspicious for impending or early rupture with measurements on the left of 5.3 × 5.8 cm and on the right of 3.9 X 3.7cm. Patient was taken urgently to the operating room and did well in the postoperative period with no complications. He was discharged from the hospital the next day.

## Discussion

Common and internal iliac artery aneurysms in isolation without concurrent abdominal aortic aneurysm occur in approximately 0.03 percent of the population. Iliac aneurysms are significantly less frequent than other aortic aneurysms, even in studies done on the asymptomatic patients.^2^ Iliac aneurysms are most common in the common iliac, followed by the internal iliac, and external iliac.^1^ Risk factors are similar to those of an abdominal aortic aneurysm, including male sex, white race, older age, history of smoking, and history of hypertension. Generally, iliac artery aneurysms have to be greater than 6.0 cm in diameter for patients to develop symptoms which can include compression, thrombosis, or thromboembolism. The average size at rupture is 5–7cm. From a surgical perspective, intervention is generally indicated for nonruptured common iliac artery aneurysms at 4cm.^2^ In a study with 63 patients, it was found that only 6.8% had rupture of an iliac artery aneurysm at less than 4 cm.^3^ Isolated iliac artery aneurysms comprise 2%–11% of intra-abdominal aneurysms.^4^

Point-of-care ultrasound is the best first line test to evaluate the aorta and iliac arteries when aneurysm or rupture is suspected. In a recent systematic review, it was shown that emergency department ultrasounds have a sensitivity of 99% and specificity of 98% for detecting abdominal aortic aneurysm.^5^ However, arteries may be difficult to evaluate if there is extensive overlying bowel gas, or if arteries are particularly deep. If possible, the iliac arteries should be visualized on ultrasound of the abdominal aorta. The visualization of the iliac arteries is not a required component of the primary ultrasound of the abdominal aorta; however, it can be included in the extended examination of the aorta.^6^ Computed tomography angiogram is next line for imaging if ultrasound is unable to identify aorta or if further evaluation is indicated.

Patients with ruptured or symptomatic aneurysms need emergent/urgent surgical repair, and most importantly, aggressive resuscitation in the emergency department. Early diagnosis is crucial for the best possible outcome. While there are clear guidelines for evaluation of abdominal aortic aneurysm, guidelines are less clear for common iliac artery aneurysm. Our case report highlights the importance of imaging past the aortic bifurcation during an ultrasound of the aorta to evaluate the iliac arteries. Similar to abdominal aortic aneurysm, rupture of the common iliac artery is a life-threatening condition and requires immediate surgical management.

## Supplementary Information














