# Supplementary figures and images for: Bilateral Common Iliac Artery Aneurysm, a Case Report
Source: J Educ Teach Emerg Med. 2020 Jan 15;5(1):V8–V11. doi: 10.21980/J83S73 (PMC10332536; doi:10.21980/J83S73)

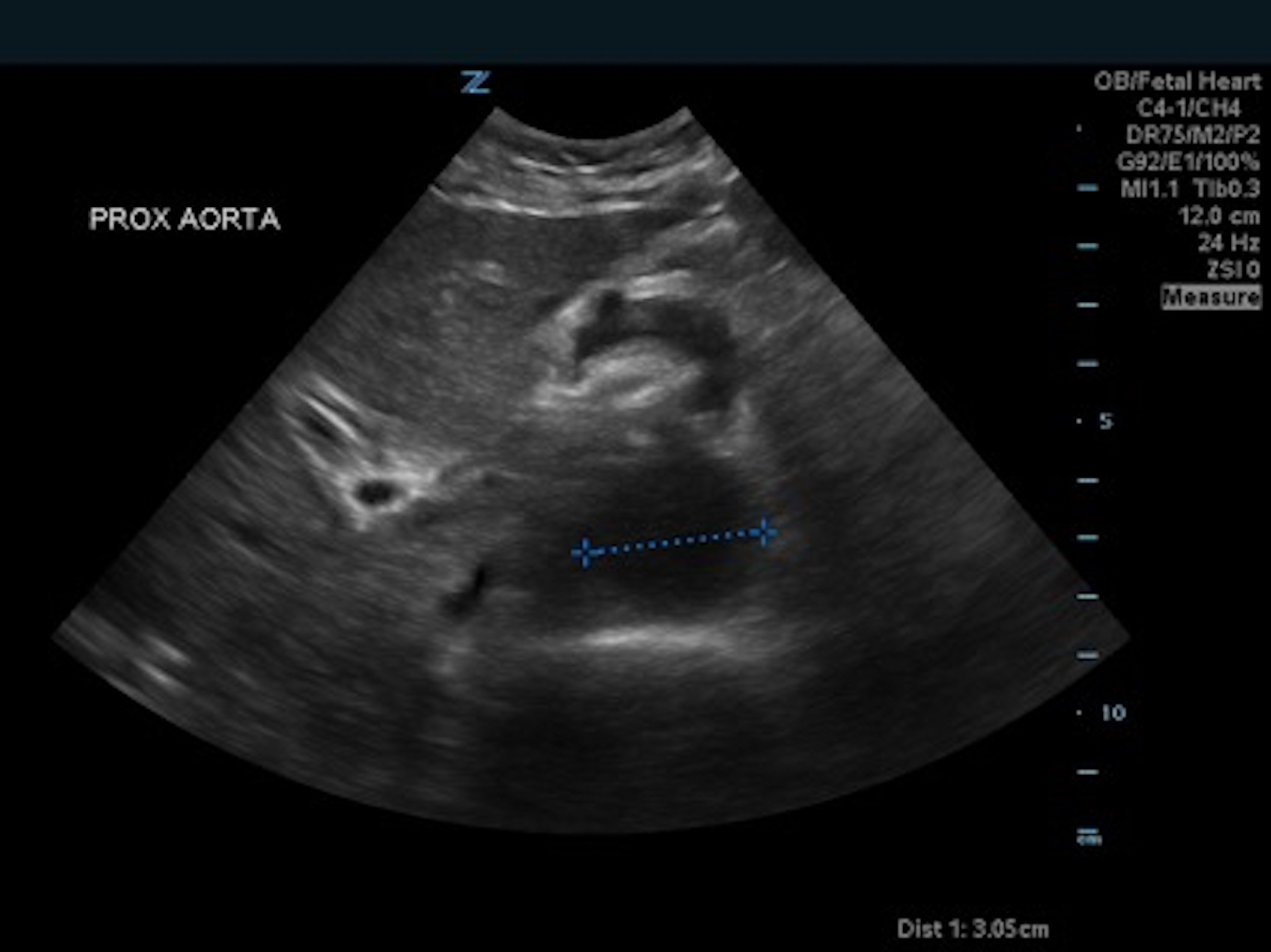

Supplement: Supplementary file 1 [file jetem-5-1-v8-supp1.jpg]

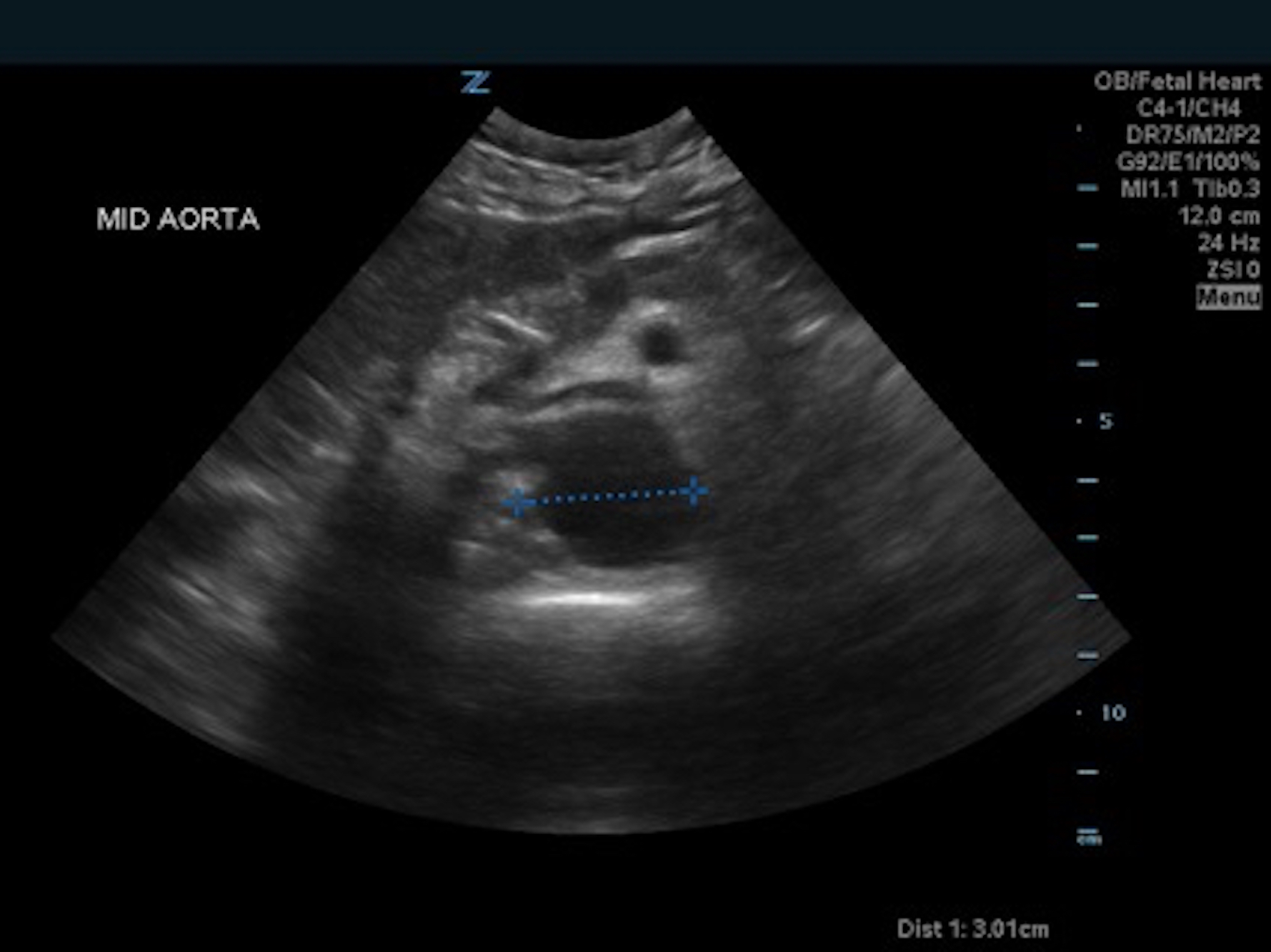

Supplement: Supplementary file 2 [file jetem-5-1-v8-supp2.jpg]

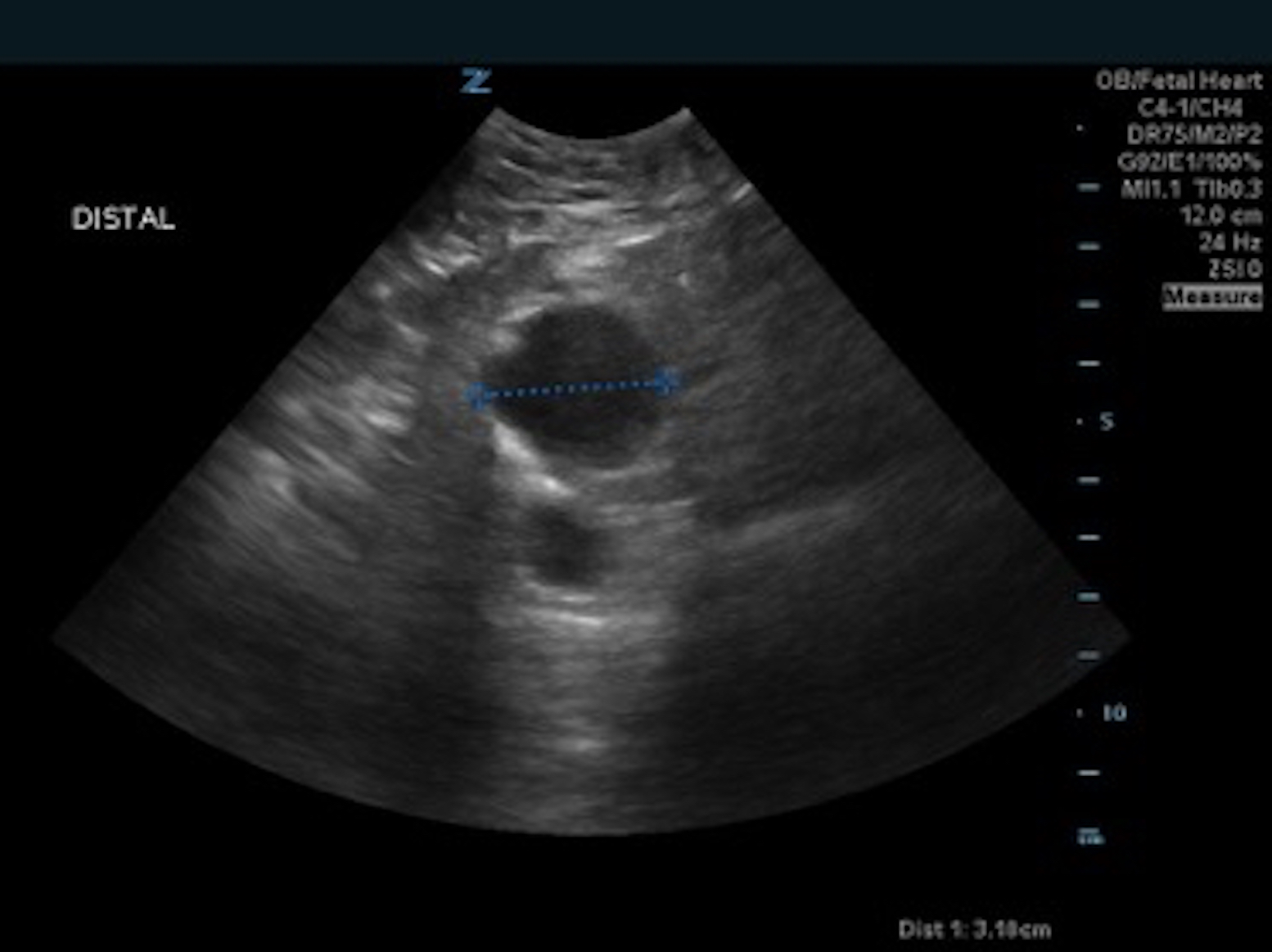

Supplement: Supplementary file 3 [file jetem-5-1-v8-supp3.jpg]

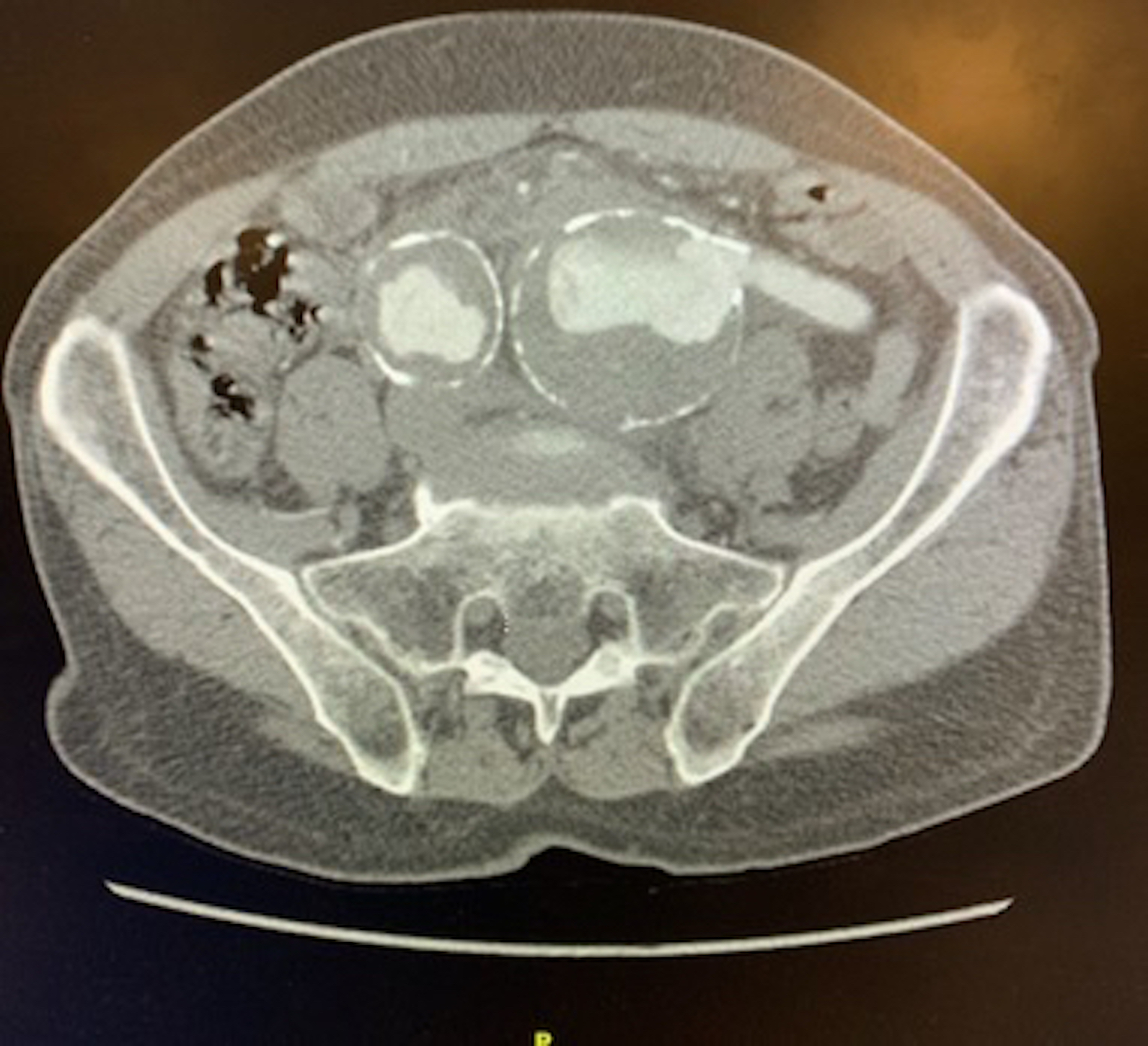

Supplement: Supplementary file 4 [file jetem-5-1-v8-supp4.jpg]

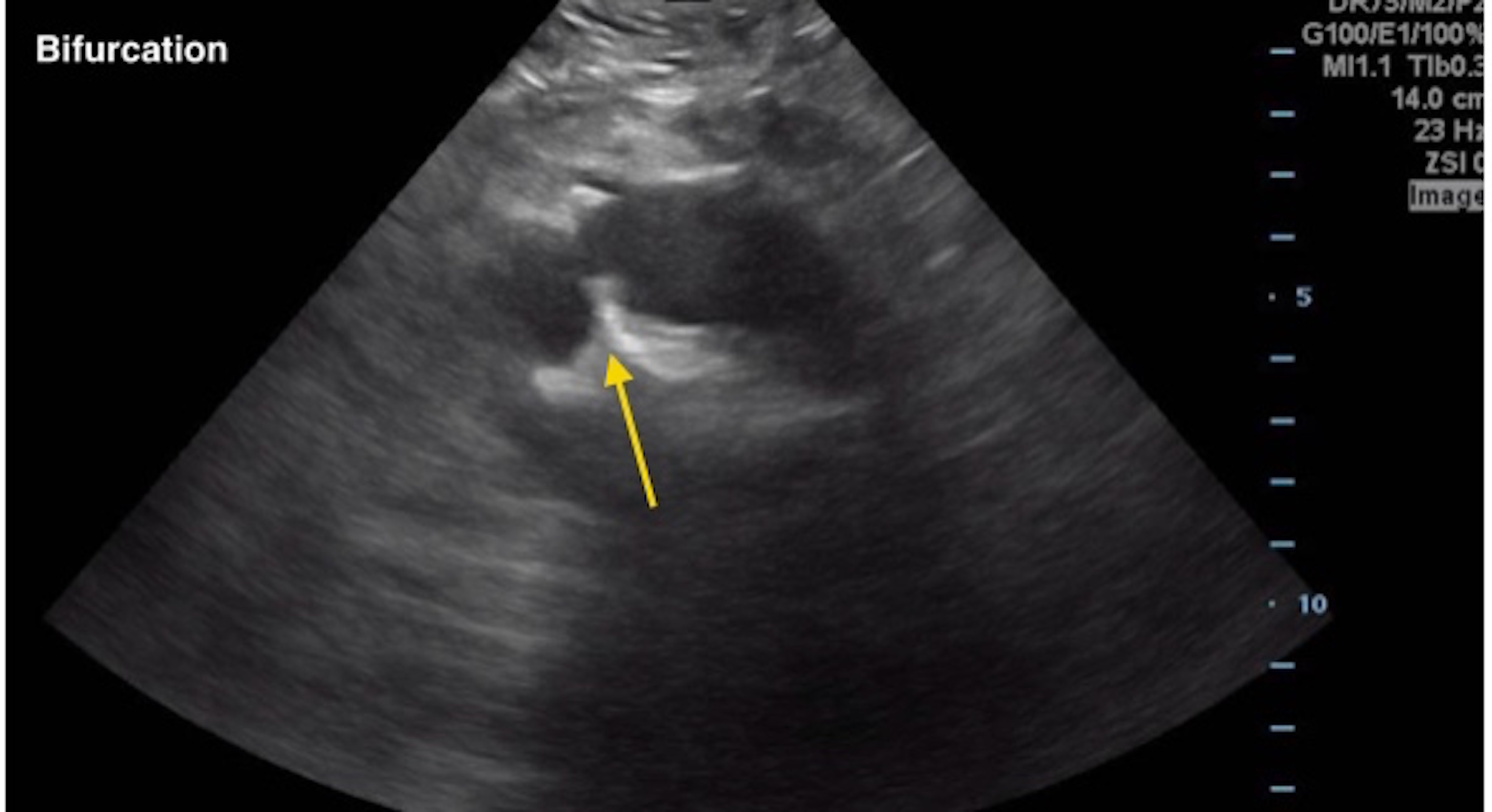

Supplement: Supplementary file 6 [file jetem-5-1-v8-supp6.jpg]

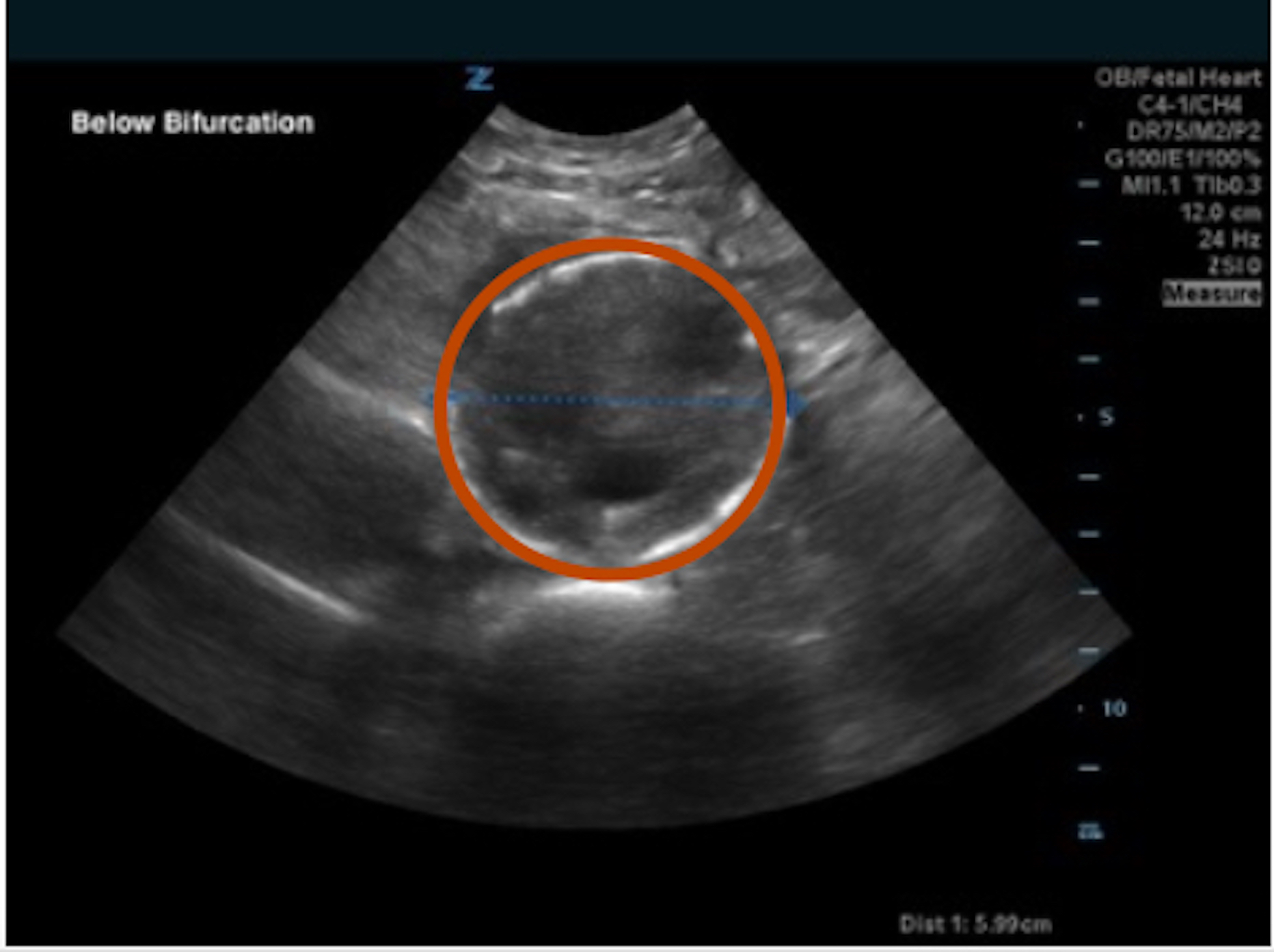

Supplement: Supplementary file 7 [file jetem-5-1-v8-supp7.jpg]
